# The clinical parameters for the diagnosis of hepatitis B virus related acute-on-chronic liver failure with sepsis

**DOI:** 10.1038/s41598-019-38866-3

**Published:** 2019-02-22

**Authors:** Ran Xue, Yueke Zhu, Hui Liu, Qinghua Meng

**Affiliations:** 1 Department of Critical Care Medicine of Liver Disease, Beijing You-An Hospital, Capital Medical University, Beijing, 100069 China; 2 Clinical-Pathology Center, Beijing You-An Hospital, Capital Medical University, Beijing, 100069 China

## Abstract

It is still unknown that whether sepsis with hepatitis B virus related acute-on-chronic liver failure (HBV-ACLF) fit into the conventional diagnostic criteria of sepsis. Our aim was to investigate the potential clinical parameters for the diagnosis of HBV-ACLF with sepsis. A retrospective study was conducted in 43 patients with HBV-ACLF and sepsis who underwent orthotopic liver transplantation. All patients were divided into three groups according to the pathological results and laboratory test results. Immunohistochemistry (IHC) staining, hematoxylin-eosin (HE) staining and Gordon Sweet’s reticulin staining were performed in this study. Alanine aminotransferase (ALT), aspartale aminotransferase (AST), total bilirubin (TBiL), cholinesterase (CHE), albumin (ALB), prothrombin activity (PTA), blood routine examination were detected. The results being chosen at admission and before transplantation were analyzed. TBiL had a significant increase (563.5 ± 191.8 umol/L vs. 383.9 ± 157.6 umol/L, 438.3 ± 154.7 umol/L, P = 0.031) and ALT significantly decreased (81.6 ± 66.4 U/L, 754.5 ± 1084.7 U/L, 120.6 ± 102.5 U/L, P = 0.005) in sepsis group before liver transplantation. When sepsis appeared in patients with HBV-ACLF, the ratio of PLT to WBC count before liver transplantation was much lower than it at admission (4.6 ± 2.0 vs. 16.1 ± 7.2, P = 0.000). In conclusion, the clinical parameters of sepsis in patients with HBV-ACLF should be reset. The ratio of PLT/WBC and (WBC_BLT_/WBC_AA_)/ (PLT_BLT_/PLT_AA_) could remind us the occurring of sepsis in patients with HBV-ACLF.

## Introduction

Acute-on-chronic liver failure (ACLF) is a life-threatening condition with a high mortality of 50–90%, defined as acute hepatic insult manifesting, such as jaundice and coagulopathy, complicated within 4 weeks by ascites and/or encephalopathy in patients with previously diagnosed or undiagnosed chronic liver disease according to the consensus recommendations of the Asian Pacific Association for the Study of the Liver (APASL)^1,2^. Hepatitis B virus (HBV) is the leading cause of chronic liver disease in the Asia-Pacific region, which is as many as 80% of the patients with ACLF in China^3,4^.

Sepsis is a common complication of ACLF, having a large effect on treatment and prognosis of ACLF^4–6^, which is one of the main causes of death^7^. Early diagnosis and treatment of sepsis are the key to improving the survival rate of patients with ACLF. Recently, sepsis has been redefined by the taskforce as a life-threatening organ dysfunction caused by a dysregulated host response to infection in the convening of the Third International Consensus Definitions Task Force of 2016^8^. However, due to impaired immunologic function in patients with ACLF, the clinical manifestation of sepsis with ACLF may not be as same as sepsis in patients without liver injury^9^. It is still unknown that whether sepsis with ACLF fit into the conventional diagnostic criteria of sepsis. Therefore, it is urgent to identify the clinical feature of sepsis with ACLF so as to perform timely and effective prevention and treatment.

Pathology has been called ‘the hidden science that saves lives’. Histopathological evaluations of biopsy tissue is the gold standard for the diagnosis of disease. In the 1980s, Lefkowitch^10^ first reported the pathological features of sepsis, that is, cholangiocholithiasis around the portal area, bile duct dilatation, and bile duct epithelial damage. These pathological features of sepsis have been recognized worldwide. As for the characteristics of HBV-ACLF with sepsis, it has been reported that massive/submassive necrosis was generated on cirrhosis with some residual nodules. There was different degree of ductular dilatation, cholestasis and epithelial cells atrophy in ductules on the margins of the residual nodules. Compared with ACLF, the distinguish feature for HBV-ACLF with sepsis is ductular cholestasis^11^.

Based on the gold standards with diagnosis of HBV-ACLF with sepsis in pathology, the aim of our study is to investigate the potential clinical parameters for the diagnosis of HBV-ACLF with sepsis.

## Material and Methods

### Study Design and Patients Selection

A retrospective study was conducted in 43 patients with HBV-ACLF and sepsis who underwent orthotopic liver transplantation from November 2004 to June 2009 in Beijing You-An Hospital, Capital Medical University. All procedures related to this research were accorded morally with current laws as well as the creeds of the Declaration of Helsinki. The research was permitted by the Ethical Committee of Beijing You-An Hospital, Capital Medical University. All study participants gave their informed consent to participate in the study.

The entry criteria comprised the following: HBV-ACLF is defined as ACLF with previously diagnosed or undiagnosed HBV. All enrolled patients met the criteria for ACLF from the consensus recommendations of the Asian Pacific Association for the Study of the Liver (APASL)^12^, and sepsis from the Third International Consensus Definitions for Sepsis and Septic Shock 2016(Sepsis-3)^8^.

The exclusion criteria were the following: other factors induce severe liver injury, such as alcohol, drugs, hepatoviruses other than HBV, autoimmunity and pregnancy, as well as genetic and metabolic disorders. HBV-ACLF patients with hepatocellular carcinoma, known decompensated cirrhosis prior to onset of acute hepatic insult, jaundice induced by hemolytic jaundice and obstructive jaundice, and prolonged prothrombin time induced by blood system diseases were also excluded.

### Hematoxylin-Eosin, Gordon Sweet’s reticulin and Immunohistochemical Staining

Immunohistochemistry (IHC) staining, routine hematoxylin-eosin(HE) staining and Gordon Sweet’s reticulin staining were performed as depicted in a previous research^13^. In detail, excised liver specimen were routinely fixed in formalin, embedded in paraffin, cut at 4-μm-thick slices, and stained with hematoxylin-eosin, Gordon Sweet’s reticulin, and immunohistochemical stains for cytokeratin 7 (Zhongshan Golden Bridge Ltd., Beijing, China). The immunohischemical staining adopted PV-6000 detector (Zhongshan Golden Bridge Ltd., Beijing, China). The staining results were assessed blindly and independently by two experienced pathologists by light microscope.

### Histological and Immunohistochemical Assessment

Pathological diagnostic criteria of sepsis were the following:^14^ Canalicular cholestasis: Bile canaliculi were dilated and contained bile thrombi in the parenchyma. Ductular cholestasis and inflammation: Bile ductular structure and canals of Hering at margins of portal tracts were dilated and filled with dense bile, and neutrophils were seen within and around the affected ductules. To analyze CK7 expression, according to the number of positive cells, the immunohistochemical results were categorized as follows: +, positive (>30%); ±, weakly positive (10–30%); −, negative (<10%).

### Laboratory Assessment

Biochemical analysis, including alanine aminotransferase (ALT), aspartale aminotransferase (AST), total bilirubin (TBiL), cholinesterase (CHE), albumin (ALB) was detected by AU5400 Automatic Biochemical Analyzer (Olympus, Japan). Blood routine examination used Sysmex-xe2100 Blood-cell counter (Sysmex, Japan). Prothrombin activity (PTA) was performed by ACLTOP automatic coagulation analyzer (Coulter, U.S.A). The results being chosen at admission and before transplantation would be analyzed.

### Statistical analysis

Statistical analysis was performed with SPSS software (Version 13.0, SPSS Inc., Chicago, IL). All results were expressed as mean (±SD). Comparisons of quantitative data were analyzed by one-way analysis of variance for multiple groups. Comparisons of white blood cell (WBC) counts and platelet (PLT) counts between at admission and before transplantation were carried out by paired t test. The differences were considered significant at P value < 0.05.

### Ethics approval and consent to participate

The investigation has been conducted in accordance with the ethical standards and according to the Declaration of Helsinki and has been approved by Beijing You-an Hospital, Capital Medical University. All study participants gave their informed consent to participate in the study.

## Results

### Baseline characteristics of patients

43 cases were collected and confirmed as HBV-ACLF. The mean age of 43 patients was 44.5 years and males were predominant (36/43, 83.7%). All patients were divided into three groups according to the pathological results and laboratory tests results. Group A: patients with sepsis included both clinical and pathological changes (n = 14). Group B: patients who had a fever (>37.5 °C) or elevated counts of WBC (≥12 × 109/L or >1.5 times the former levels) but no pathological changes of sepsis(n = 10). Group C: patients without sepsis included both clinical and pathological changes (n = 19).

As we can see from Table 1, there was no significant difference in average onset age among three groups. The course from start to liver transplantation in group A was much longer than group B and C (33.4 ± 24.2 days vs. 13.6 ± 9.3 days, 10.8 ± 7.6 days) (P = 0.000). The ascitic fluid had positive culture only in one patient and the blood culture was positive in two patients in group A which developed a urinary tract infection and pneumonia respectively.

**Table 1:** Comparison of clinical and laboratory test result among three groups.

N = 43	Group A (n = 14)	Group B (n = 10)	Group C (n = 19)	F	p value
Age	46.2 ± 12.1	46.4 ± 13.5	42.2 ± 8.7	0.718	0.494
Course from start to transplantation	33.4 ± 24.2	13.6 ± 9.3*	10.8 ± 7.6*	9.399	0.000
ALT _AA_ (U/L)	369.3 ± 413.1	923.7 ± 699.0	429.2 ± 709.3	2.684	0.081
ALT _BLT_ (U/L)	81.6 ± 66.4	754.5 ± 1084.7*	120.6 ± 102.5#	6.015	0.005
AST _AA_ (U/L)	252.2 ± 233.3	624.9 ± 563.2	465.4 ± 771.1	1.185	0.316
AST _BLT_ (U/L)	86.6 ± 32.4	278.5 ± 232.5*	181.7 ± 222.1	3.129	0.055
TBIL _AA_ (umol/L)	422.9 ± 183.5	318.4 ± 135.1	328.7 ± 125.7	2.051	0.142
TBIL _BLT_ (umol/L)	563.5 ± 191.8	383.9 ± 157.6*	438.3 ± 154.7*	3.800	0.031
PTA% _AA_	29.0 ± 10.8	26.9 ± 10.4	28.7 ± 10.2	0.129	0.879
PTA% _BLT_	29.2 ± 13.1	21.6 ± 10.9	24.9 ± 9.9	1.382	0.263
CHE _AA_ (U/L)	3174.7 ± 1247.0	3527.5 ± 970.8	2967.8 ± 1171.4	0.769	0.470
CHE _BLT_ (U/L)	3140.0 ± 1584.8	3127.4 ± 1390.3	2705.7 ± 1082.5	0.549	0.582
ALB _AA_ (g/L)	31.0 ± 5.1	31.7 ± 4.3	29.8 ± 3.5	0.610	0.548
ALB _BLT_ (g/L)	32.4 ± 5.3	32.9 ± 3.2	30.0 ± 3.5	2.032	0.144
WBC _AA_ (×10^9^/L)	6.7 ± 4.7	8.8 ± 3.5	5.0 ± 1.6^#^	4.316	0.020
WBC_BLT_ (×10^9^/L)	11.2 ± 4.5	9.4 ± 4.2	7.0 ± 8.7	1.616	0.211
WBC_BLT_/WBC _AA_	2.3 ± 1.6	1.1 ± 0.6*	1.4 ± 1.5	2.854	0.069
PLT_AA_ (×10^9^/L)	83.9 ± 41.1	118.8 ± 53.6	79.6 ± 45.7	2.542	0.091
PLT_BLT_ (×10^9^/L)	48.1 ± 20.8	109.8 ± 52.2*	74.7 ± 38.0^#^	7.910	0.001
PLT_BLT_/PLT _AA_	0.7 ± 0.3	0.9 ± 0.2*	1.0 ± 0.2*	8.721	0.001
PLT_AA_/WBC_AA_	16.1 ± 7.2	15.2 ± 8.5	16.4 ± 9.3	0.066	0.937
PLT_BLT_/WBC_BLT_	4.6 ± 2.0	12.5 ± 4.9*	14.9 ± 8.6*	11.28	0.000
(WBC_BLT_/WBC _AA_)/(PLT_BLT_/PLT _AA_)	3.9 ± 2.0	1.2 ± 0.6*	1.5 ± 1.8*	10.475	0.000

AA: at admission; BLT: before liver transplantation.

*Comparison with group A, p < 0.05; ^#^Comparison with group B, p < 0.05.

### Histopathological features of patients

**Figure 1:**
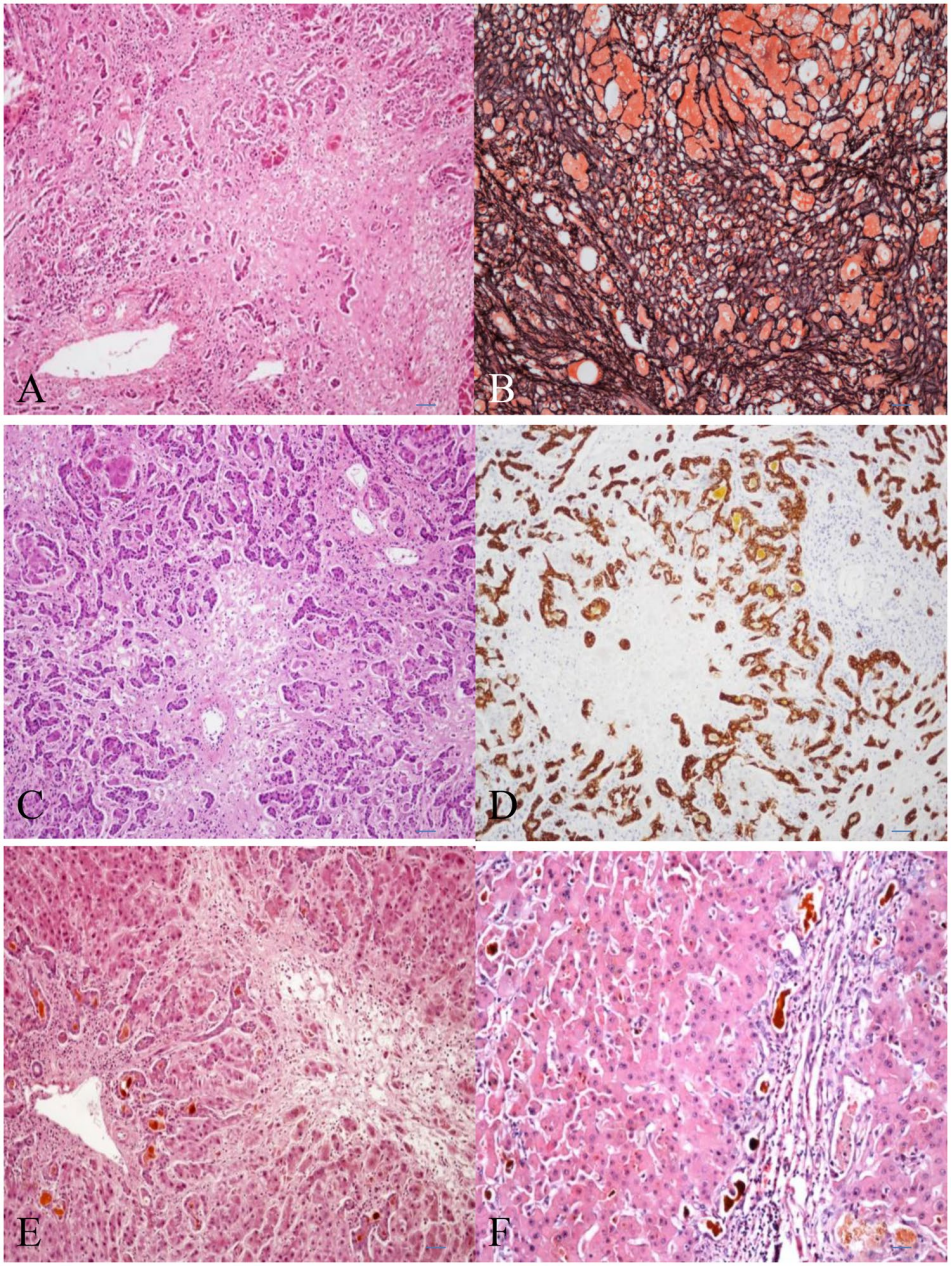
(**A**) Massive hepatic necrosis, liver cell dropout, residual hepatocytes and intact portal tract pattern could be seen in this field (HE; original magnification, x100). (**B**) Part reticular frameworks reserved, and other had already collapsed (Gordon-Sweet reticulin; original magnification, x100). (**C**) Massive hepatic necrosis, prominent ductular proliferation was in this field (HE original magnification, x100); (**D**) Cytokeratin7 immunostaining shows proliferating bile ductile (Immunohistochemistry; original magnification, x100). (**E**) Bile ductular structures were dilated and contain inspissated bile at the margins of portal tract. Liver cell dropout occurred around a terminal hepatic venule (HE; original magnification, x100); (**F**) Proliferated bile ductules around the regenerating nodules were dilated and contained inspissated bile. The ductular epithelium became atrophic and distortional (HE; original magnification, x100).

All cases showed features of chronic hepatitis B. Massive or submassive hepatic necrosis and liver cell dropout appeared in the background of chronic hepatitis B or cirrhosis (Fig. 1A). Reticular frameworks either reserved or had already collapsed (Fig. 1B). The portal-perivenular architectural pattern remained intact. A prominent ductular reaction was seen with associated neutrophils or other inflammatory cell infiltration (Fig. 1C,D). Regenerating nodules of hepatocytes, which are often bile stained, might be irregularly distributed in the liver.

14 of all cases showed that proliferated bile ductules at the edge of the portal tract or around the regenerating nodules were dilated and contained inspissated bile (Fig. 1E,F). Accordingly, the ductular epithelium became atrophic and distortional.

As for the detailed differences between group A and B in histological characteristics, all patients in group A had the distinctive characteristics with a serials of dilated canaliculi with atrophy epithelium and coarse bile plugs arranged in clusters around the regeneration node, while in group B, only a little proportion of patients had these characteristics, these differences were very marked significance (P < 0.005) (Table 2).

**Table 2:** Summary for the histological characteristics in HBV-ACLF with sepsis.

Histopathological changes	Group A	Group B	P value
Scope of hepatocellular necrosis			
<30%	3 (33.3%)	3 (30%)	0.928
30–60%	4 (44.4%)	4 (40%)	
>60%	2 (22.2%)	3 (30%)	
Ductular reaction			
None	0	1 (10.0%)	0.105
Mild	0	3 (30.0%)	
Moderate	4 (44.44%)	3 (30.0%)	
Marked	5 (55.56%)	3 (30.0%)	
Ductular cholestasis			
with	9 (100%)	6 (60.0%)	0.087
without	0	4 (40.0%)	
hepatocellular swelling			
None	5 (55.56%)	4 (40.0%)	0.763
Mild	1 (11.11%)	2 (20.0%)	
Moderate	0	0	
Marked	3 (33.33%)	4 (40.0%)	
Bile plugs in canalici			
None	2 (22.22%)	3 (30.0%)	0.131
Mild	2 (22.22%)	2 (20.0%)	
Moderate	0	3 (30.0%)	
Marked	5 (55.56%)	2 (20.0%)	
Bile plugs in ductul around regenerative nodules			
None	0	4 (40.0%)	0.000
Mild	1 (11.11%)	6 (60.0%)	
Moderate	8 (88.89%)	0	
Marked	0	0	
Ductular epithelium atrophy			
with	9 (100%)	2 (20.0%)	0.001
without	0	8 (80.0%)	

### The clinical serum parameters of pantients

As shown in Table 1, the serum ALT, AST, TBiL, CHE, ALB and PTA among three groups had no significant difference at admission. Only TBiL had a significant increase (563.5 ± 191.8 umol/L vs. 383.9 ± 157.6 umol/L, 438.3 ± 154.7 umol/L, P = 0.031) and ALT significantly decreased (81.6 ± 66.4 U/L, 754.5 ± 1084.7 U/L, 120.6 ± 102.5 U/L, P = 0.005) in group A before liver transplantation. These results were consistent with the manifestation of biliary enzyme separation.

**Figure 2:**
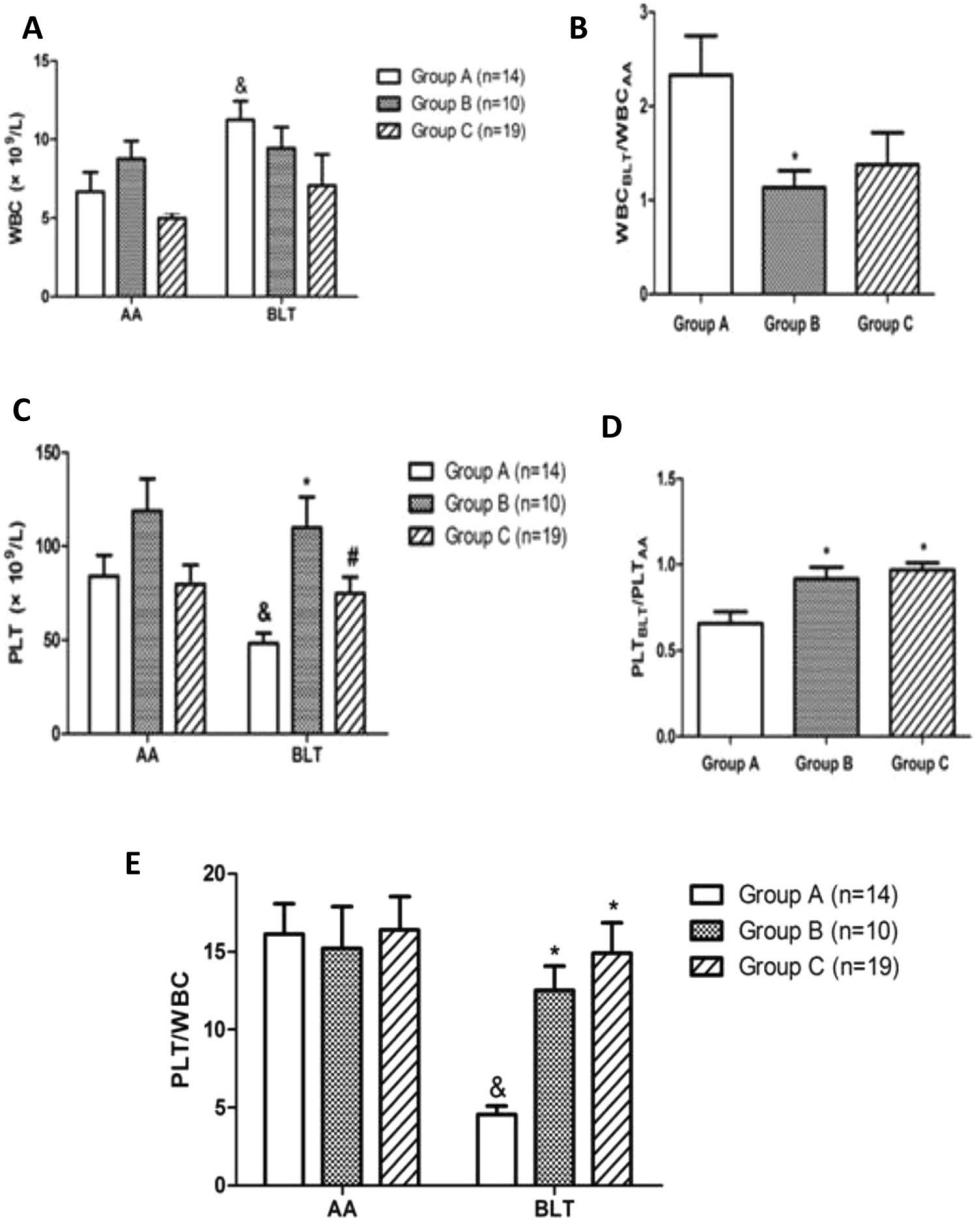
(**A**) WBC count at admission in group A was lower than group B, but it was higher than group B before liver transplantation. ‘&’: WBC count before liver transplantation was much higher than it at admission in group A (P < 0.05). (**B**) The degree of elevation of WBC count in group A was much higher than it in other groups(P < 0.05). (**C**) PLT count before liver transplantation in group A was much lower than group B and C (P < 0.01). In group A, PLT count before liver transplantation was much lower than it at admission (P < 0.05). (**D**) The degree of decrease of PLT count in group A was much more than it in other groups (P < 0.05). (**E**) The ratio of PLT /WBC before liver transplantation in group A was much lower than it in other groups (P < 0.01). The ratio before liver transplantation in group A was also much lower than it at admission (P < 0.01).

Although the WBC count at admission in group A was lower than group B, its increased much more than group B before liver transplantation (P < 0.05) (Fig. 2A,B). The PLT before liver transplantation in group A was much lower than group B and C (48.1 ± 20.8 × 109/L vs. 109.8 ± 52.2 × 109/L, 74.7 ± 38.0 × 109/L, P = 0.001). However, the PLT count in the three groups had no statistical difference at admission (P = 0.091) (Fig. 2C,D).

The ratio of PLT to WBC before liver transplantation in group A was the significantly lower than other two groups (4.6 ± 2.0, 12.5 ± 4.9, 14.9 ± 8.6, respectively, P = 0.000) (Fig. 2E). (WBC_BLT_/WBC _AA_)/(PLT_BLT_/PLT _AA_) also revealed a remarkable increase of WBC count and decrease of PLT count in group A before liver transplantation(3.9 ± 2.0, 1.2 ± 0.6, 1.5 ± 1.8, P = 0.000).

Furthermore, we compared PLT, WBC count and their ratio at admission with before liver transplantation in each group respectively. A significant difference for above three parameters was detected only in group A. In group A, PLT before liver transplantation reduced much more than it at admission (48.1 ± 20.8 × 109/L vs. 83.9 ± 41.1 × 109/L, P = 0.01). On the contrary, WBC count elevated significantly before liver transplantation (11.2 ± 4.5 × 109/L vs. 6.7 ± 4.7 × 109/L, P = 0.004). The ratio of PLT to WBC count before liver transplantation was also much lower than it at admission (4.6 ± 2.0 vs.16.1 ± 7.2, P = 0.000) (Table 3).

**Table 3:** Comparison of PLT and WBC count between at admission and before liver transplantation in each group.

N = 43	Group A (N = 14)	p value	Group B (N = 10)	p value	Group C (N = 19)	p value
PLT(×10^9^/L)						
AA	83.9 ± 41.1	0.01*	118.8 ± 53.6	0.22	79.6 ± 45.7	0.402
BLT	48.1 ± 20.8		109.8 ± 52.2		74.7 ± 38.0	
WBC(×10^9^/L)						
AA	6.7 ± 4.7	0.004*	8.8 ± 3.5	0.573	5.0 ± 1.6	0.299
BLT	11.2 ± 4.5		9.4 ± 4.2		7.0 ± 8.7	
PLT/WBC						
AA	16.1 ± 7.2	0.000*	15.2 ± 8.5	0.301	16.4 ± 9.3	0.435
BLT	4.6 ± 2.0		12.5 ± 4.9		14.9 ± 8.6	

AA: at admission; BLT: before liver transplantation.

*P < 0.01, remarkable significance.

## Discussion

This is the first retrospective study about clinical parameters analysis of sepsis with HBV-ACLF based on histopathological changes. Recently, sepsis has been defined as life-threatening organ dysfunction caused by a dysregulated host response to infection^8^. However, its diagnostic criteria including general variables, inflammatory variables, hemodynamic variables, organ dysfunction variables, tissue perfusion variables is not suitable for patients in HBV-ACLF with sepsis, because patients with underlying chronic liver disease and cirrhosis may have deranged clinical parameters. It leads to many difficulties in clinical diagnosis of HBV-ACLF with sepsis according to the conventional diagnostic criteria of sepsis. Therefore, it’s essential to establish compatible diagnostic criteria of sepsis in patients with ACLF.

Histopathological evaluations of tissue is the gold standard for the diagnosis of disease. In 1979, Zimmerman, *et al*. proposed canalicular cholestasis was the most common pattern of hepatic changes in sepsis^15^. In 1982, Banks and Lefkowitch^10,16^ respectively described another patterns of sepsis–bile ductular cholestasis and inflammation which showed proliferated bile ductules at the margins of portal tracts dilated and contained bile. Meanwhile, there usually exist perivenular cholestasis. Koskinas, *et al*.^17^ summarized the liver histology in 15 ICU patients dying from sepsis, including Zone 3 necrosis, canalicular cholestasis, bile ductular cholestasis, cholangitis, cholangiolitis, and steatosis. YANG Shu-yin *et al*. reported that the characteristic of HBV-ACLF with sepsis was ductular cholestasis, which should be distinguished from other types of cholestasis^11^. All above liver histological studies provided us a basic foundation for pathologic diagnosis of ACLF with sepsis.

In our study, the serum TBiL level was an undoubtedly important indicator in observed biochemical parameters. We found TBiL level elevated significantly in HBV-ACLF with sepsis group before liver transplantation (P < 0.05), although it also elevated in other two groups. The patients with ACLF often have hyperbilirubinemia because of massive hepatic necrosis. Hyperbilirubinemia is also one of the main performances in sepsis and it can occur via multiple mechanisms, such as hemolysis, hepatic dysfunction, cholestasis^18^. We inferred that sepsis might appear if the serum TBiL level of patients with HBV-ACLF remarkably elevated during the course of illness.

The WBC count has always been regarded as an infection indicator. The count >12000 × 10^9^/L used to be one of the diagnostic criteria of sepsis^19^. However, the WBC count is usually low in patients with chronic liver disease especially cirrhosis. In our study, the WBC count was only 6.7 ± 4.7 × 10^9^/L in the patients with sepsis group at admission, which was lower than in group B (8.8 ± 3.5 × 10^9^/L). However, before liver transplantation, the WBC count markedly elevated in the patients with sepsis group (11.2 ± 4.5 × 10^9^/L vs. 6.7 ± 4.7 × 10^9^/L, P = 0.004). And the degree of elevation (WBC_BLT_/WBC_AA_) was much higher than it in group B (P < 0.05). Therefore, our study suggested that ever higher WBC counts might be an indicator of sepsis occurring in patients with ACLF, although positive rate of blood and ascitic fluid culture were very low, which was in accord with the study of Garg H *et al*. They found that the result of ascitic fluid culture was positive in 7% patients and blood culture was positive only in 4% patients. And they also regarded high WBC counts as an infection indicator^20^.

Thrombocytopenia (PLT < 100,000 × 10^9^/L) used to be also considered one of diagnostic criteria of sepsis^19^. A lower PLT count was observed in patients with sepsis due to endothelial damage, production of cytokines, and bone marrow suppression^21,22^. In our study, patients with HBV-ACLF had a decreased PLT count because of their underlying chronic liver disease. However, the PLT count declined much more remarkably in sepsis group before liver transplantation than it at admission (48.1 ± 20.8 × 10^9^/L vs. 83.9 ± 41.1 × 10^9^/L, P = 0.004), and it was much lower than other two group before liver transplantation (P < 0.01). The value of PLT_BLT_/PLT_AA_, which represented the degree of decrease from at admission to before liver transplantation, also indicated PLT count exactly decreased when sepsis occurred. We considered that marked decrease of PLT count might be used to predict sepsis appearing in patients with HBV-ACLF.

In addition, we calculated the ratio of PLT/WBC and (WBC_BLT_/WBC_AA_)/(PLT_BLT_/PLT_AA_). Both of them further revealed that from at admission to before liver transplantation, WBC count increased and PLT count decreased significantly in sepsis with HBV-ACLF. Based on our data, the ratio of PLT/WBC and (WBC_BLT_/WBC_AA_)/(PLT_BLT_/PLT_AA_) can indicate the occurrence of sepsis in HBV-ACLF.

In our study, it was interesting that the course from start to liver transplantation in sepsis group was much longer than that of other two groups(P < 0.01). Until now, there is no study showed the relationship between the course of illness and sepsis occurring. Could we have a conclusion that the longer was the course of illness, the greater was the risk of sepsis? Whether we could consider that sepsis might be a complication rather than the initial precipitating event in patients with ACLF in accordance with our data? We needed further investigation with a larger number of patients.

There was one case with positive pathological diagnosis but no clinical manifestations of sepsis which is not included in the analysis of 43 patients. That may be related to the serious body condition of patients whose immune response is very low so that cannot resist the pathogen. Clinical manifestations such as fever and abdominal pain are mostly caused by the immune system resisting pathogens and releasing inflammatory factors. Therefore, pathological examination is of great significance in diagnosing such HBV-ACLF patients with sepsis who don’t have obvious symptoms of sepsis.

This study has some limitations. Because it is tough to collect more patients which have pair of tissue pathological and clinical result, a larger sample size is still needed to validate our findings in the future.

## Conclusions

The clinical parameters of sepsis in patients with HBV-ACLF should be reset. When sepsis appeared, the serum TBiL level and WBC count remarkably elevated, while PLT count significantly decreased. The ratio of PLT/WBC and (WBC_BLT_/WBC_AA_)/(PLT_BLT_/PLT_AA_) could remind us the occurring of sepsis in patients with HBV-ACLF.

## References

[1] Sarin SK (2009). Acute-on-chronic liver failure: consensus recommendations of the Asian Pacific Association for the study of the liver (APASL). Hepatol Int.

[2] Katoonizadeh A (2010). Early features of acute-on-chronic alcoholic liver failure: a prospective cohort study. Gut.

[3] Du WB (2005). Effects of artificial liver support system on patients with acute or chronic liver failure. Transplant Proc..

[4] Xue R (2017). A novel dynamic model for predicting outcome in patients with hepatitis B virus related acute-on-chronic liver failure. Oncotarget..

[5] Yuen M-F (2003). Role of hepatitis B virus genotypes in chronic hepatitis B exacerbation. Clin. Infect. Dis..

[6] Omata M (1991). Mutations in the precore region of hepatitis B virus DNA in patients with fulminant and severe hepatitis. N. Engl. J. Med..

[7] Fan HL (2012). Predictors of the outcomes of acute-on-chronic hepatitis B liver failure. World J Gastroenterol..

[8] Singer M (2016). The Third International Consensus Definitions for Sepsis and Septic Shock (Sepsis-3). JAMA..

[9] Jha AK, Nijhawan S, Suchismita A (2011). Sepsis in acute on chronic liver failure. Dig Dis Sci..

[10] Lefkowitch JH (1982). Bile ductular cholestasis: an ominous histopathologic sign related to sepsis and “cholangitis lenta”. Hum Pathol..

[11] YANG Shu-yin LI, Shu-ting XUE (2014). Feng,et al. Pathological characteristics of hepatitis B virus related acute-on chronic liver failure complicated with sepsis. Chinese Journal of Hepatology (Electronic Edition)..

[12] Sarin SK (2014). Acute-on-chronic liver failure: consensus recommendations of the Asian Pacific Association for the Study of the Liver (APASL) 2014. Hepatol. Int..

[13] Xue R (2017). The significance of glypican-3 expression profiling in the tumor cellular origin theoretical system for hepatocellular carcinoma progression. J Gastroenterol Hepatol..

[14] Lefkowitch J. H. Scheuer’s Liver Biopsy Interpretation, 8th ed. New York: Saunders, 331–332 (2010).

[15] Zimmerman HJ (1979). Jaundice due to bacterial infection. Gastroenterology.

[16] Banks JG (1982). Liver function in septic shock. J Clin Pathol.

[17] Koskinas J (2008). Liver histology in ICU patients dying from sepsis: A clinico-pathological study. World J Gastroenterol.

[18] Chand N, Sanyal AJ (2007). Sepsis-induced cholestasis. Hepatology..

[19] Dellinger R. P. *et al*. Surviving Sepsis Campaign: international guidelines for management of severe sepsis and septic shock, 2012. *Intensive Care Med*. 2013 Feb; **39**(2), 165–228. 10.1007/s00134-012-2769-8 PMC7095153 23361625

[20] Garg H (2012). Clinical profile and predictors of mortality in patients of acute-on-chronic liver failure. Dig Liver Dis..

[21] Guclu E, Durmaz Y, Karabay O (2013). Effect of severe sepsis on platelet count and their indices. Afr Health Sci..

[22] Aydemir H (2015). Platelet and mean platelet volume kinetics in adult patients with sepsis. Platelets..

